# Supplementary material for: Differential expression of NF-κB heterodimer RelA/p50 in human urothelial carcinoma
Source: PeerJ. 2018 Sep 13;6:e5563. doi: 10.7717/peerj.5563 (PMC6139250; doi:10.7717/peerj.5563)
Supplement: Supplemental Information 1 [file peerj-06-5563-s001.docx]

**Proteins of the NFKB family along with respective UniProt IDs**

| **Family Proteins** | **Uniprot ID** |
| --- | --- |
| NFkB1 | P19838 |
| NFkB3/RELA | Q04206 |
| RELB | Q01201 |
| NFKB2/p100 | Q00653 |
| IkBalpha | P25963 |
| IkappaBbeta | Q15653 |
| IkappaBepsilon | O00221 |
| BCL3 | P20749 |
| IKKalpha/CHUK | O15111 |
| IKKbeta/IKBKB | O14920 |
| IKBK gamma (NEMO) | Q9Y6K9 |
