# Supplementary material for: Differential expression of NF-κB heterodimer RelA/p50 in human urothelial carcinoma
Source: PeerJ. 2018 Sep 13;6:e5563. doi: 10.7717/peerj.5563 (PMC6139250; doi:10.7717/peerj.5563)
Supplement: Supplemental Information 2 [file peerj-06-5563-s002.docx]

**Targets of the NFKB family proteins as retrieved from KEGG pathway along with its respective UniProt IDs**

| **Target Proteins** | **Uniprot ID** |
| --- | --- |
| ICAM1 | P05362 |
| TRAF1 | Q13077 |
| VEGFA | P15692 |
| BAFF | Q9Y275 |
| cIAP-1 | Q13490 |
| cIAP-2 | Q13489 |
| XIAP | P98170 |
| cFLIP | O15519 |
| Bcl-2 | P10415 |
| Bcl-xL | Q92934 |
| TRAF2 | Q12933 |
| Bfl-1 | Q16548 |
| IL-8 | P10145 |
| IL-1beta | P01584 |
| TNFalpha | P01375 |
| COX2 | P35354 |
| MIP-1beta | P13236 |
| MIP-2 | P19875 |
| VCAM-1 | P19320 |
| MMP-9 | P14780 |
| manganese superoxide dismutase | P04179 |
| IEX-1L | O75353 |
| cyclinD1 | P24385 |
| c-Myc | P01106 |
