# Supplementary figures and images for: Differential expression of NF-κB heterodimer RelA/p50 in human urothelial carcinoma
Source: PeerJ. 2018 Sep 13;6:e5563. doi: 10.7717/peerj.5563 (PMC6139250; doi:10.7717/peerj.5563)

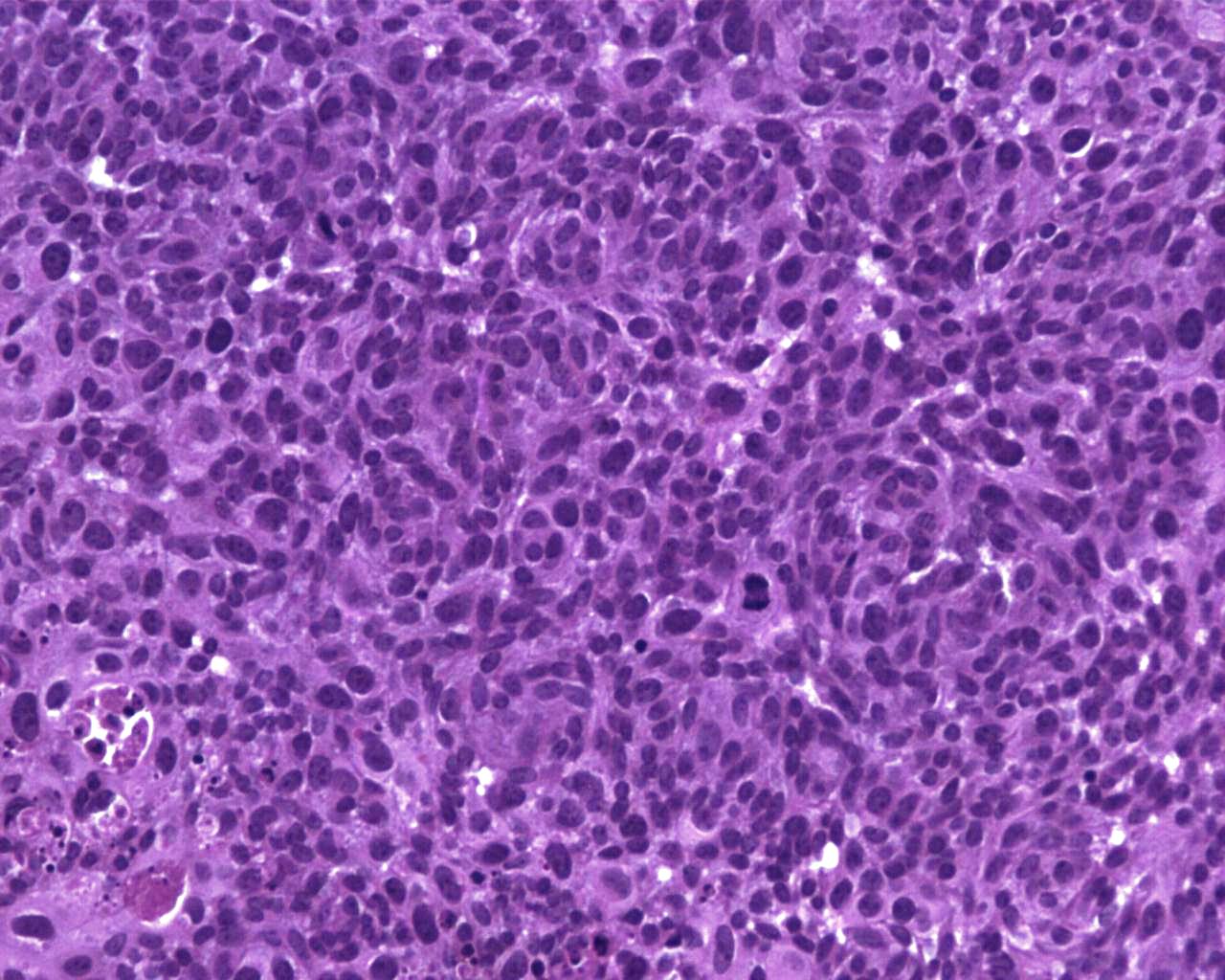

Supplement: Supplemental Information 3 — (A) High grade non invasive urothelial carcinoma H&E × 200. [file peerj-06-5563-s003.jpg]

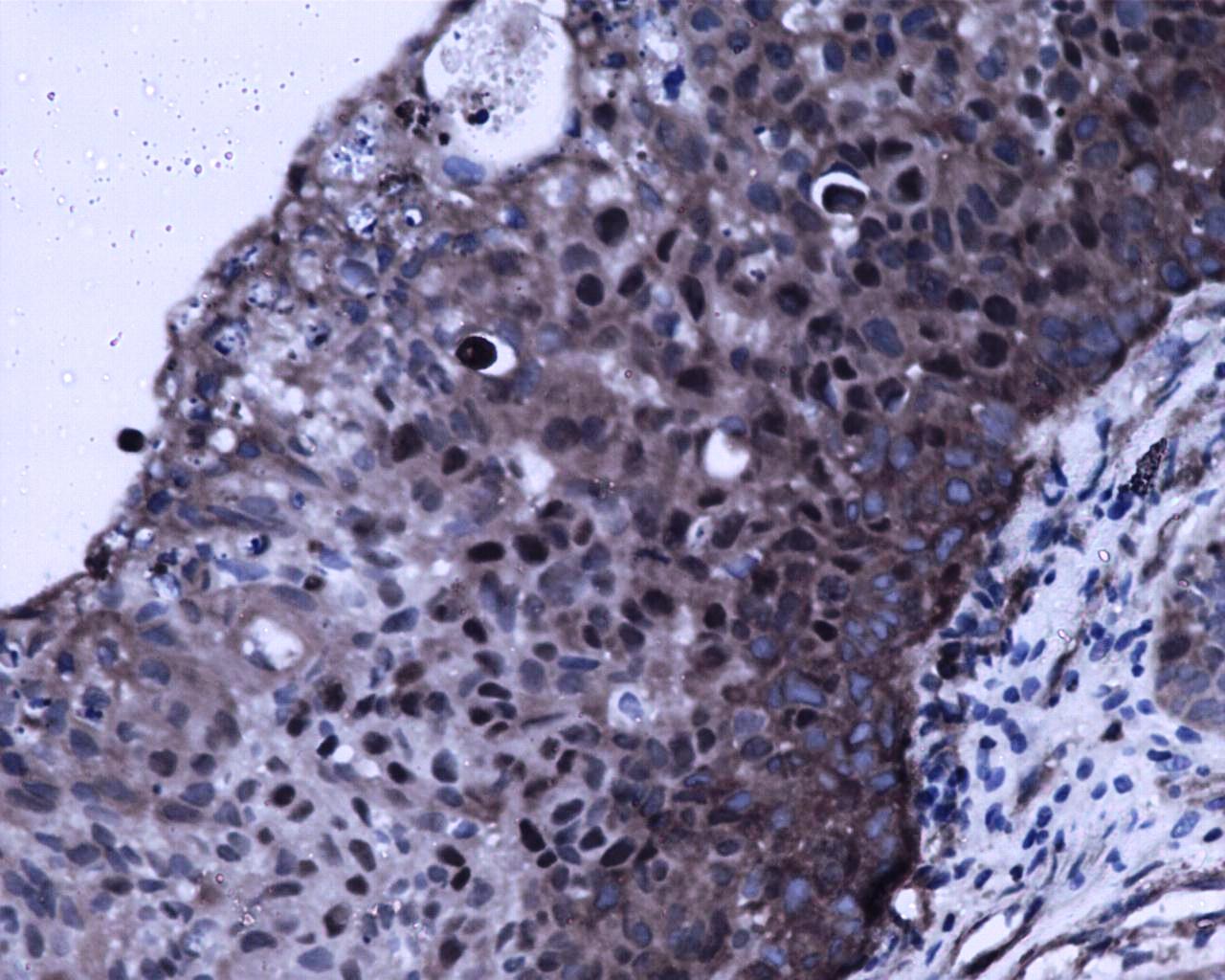

Supplement: Supplemental Information 4 — (B) Immunostaining with RelA (p65) showed nuclear positivity (Brown colour) indicated by arrow IHC × 200. [file peerj-06-5563-s004.jpg]

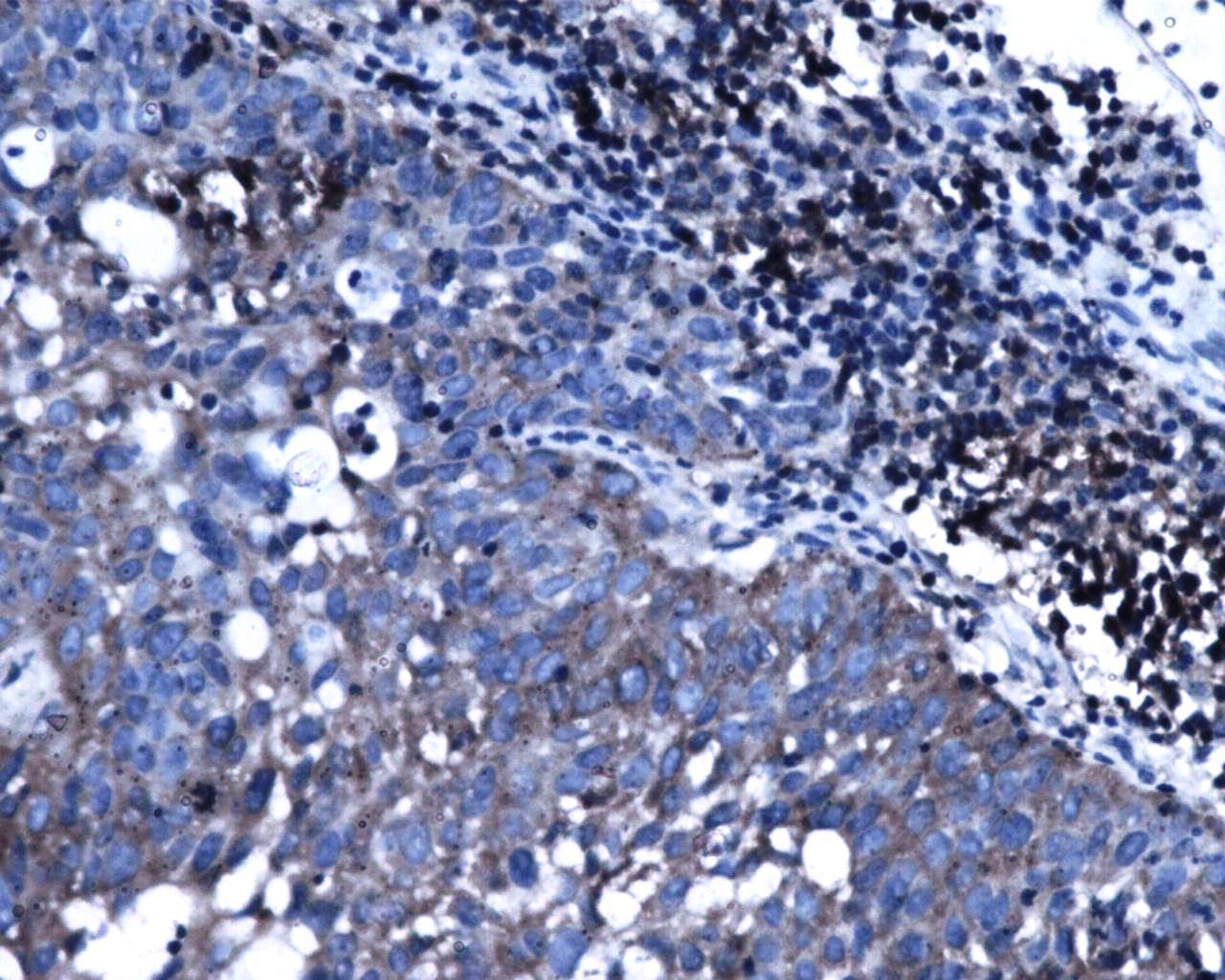

Supplement: Supplemental Information 5 — (C) NF-κB p50 showed moderate cytoplasmic positivity IHC × 200. Note: adjacent lymphocytes (in-built control) have taken up nuclear staining indicated by arrows. [file peerj-06-5563-s005.jpg]

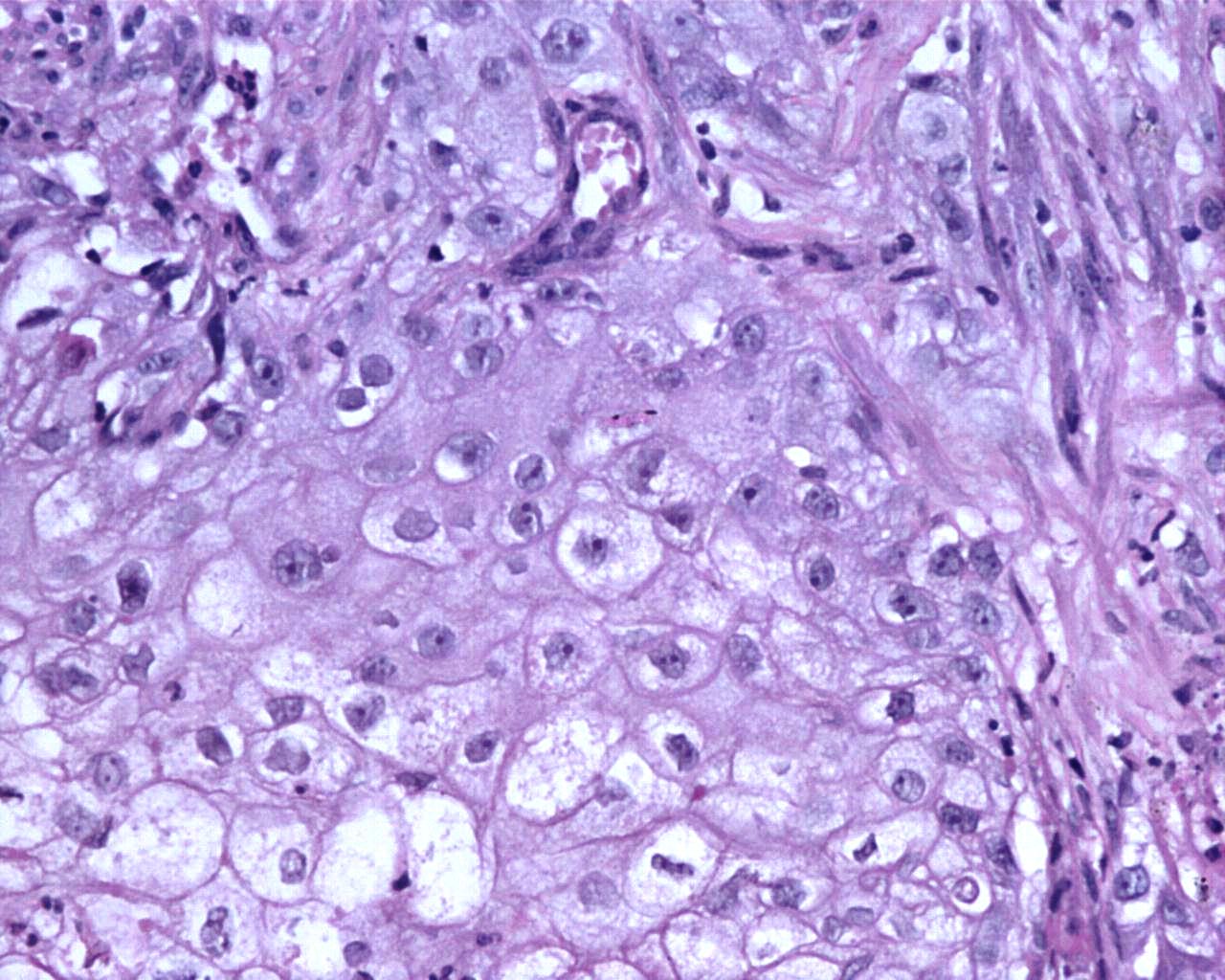

Supplement: Supplemental Information 6 — (D) High grade papillary invasive urothelial carcinoma with squamous differentiation H&E × 200. [file peerj-06-5563-s006.jpg]

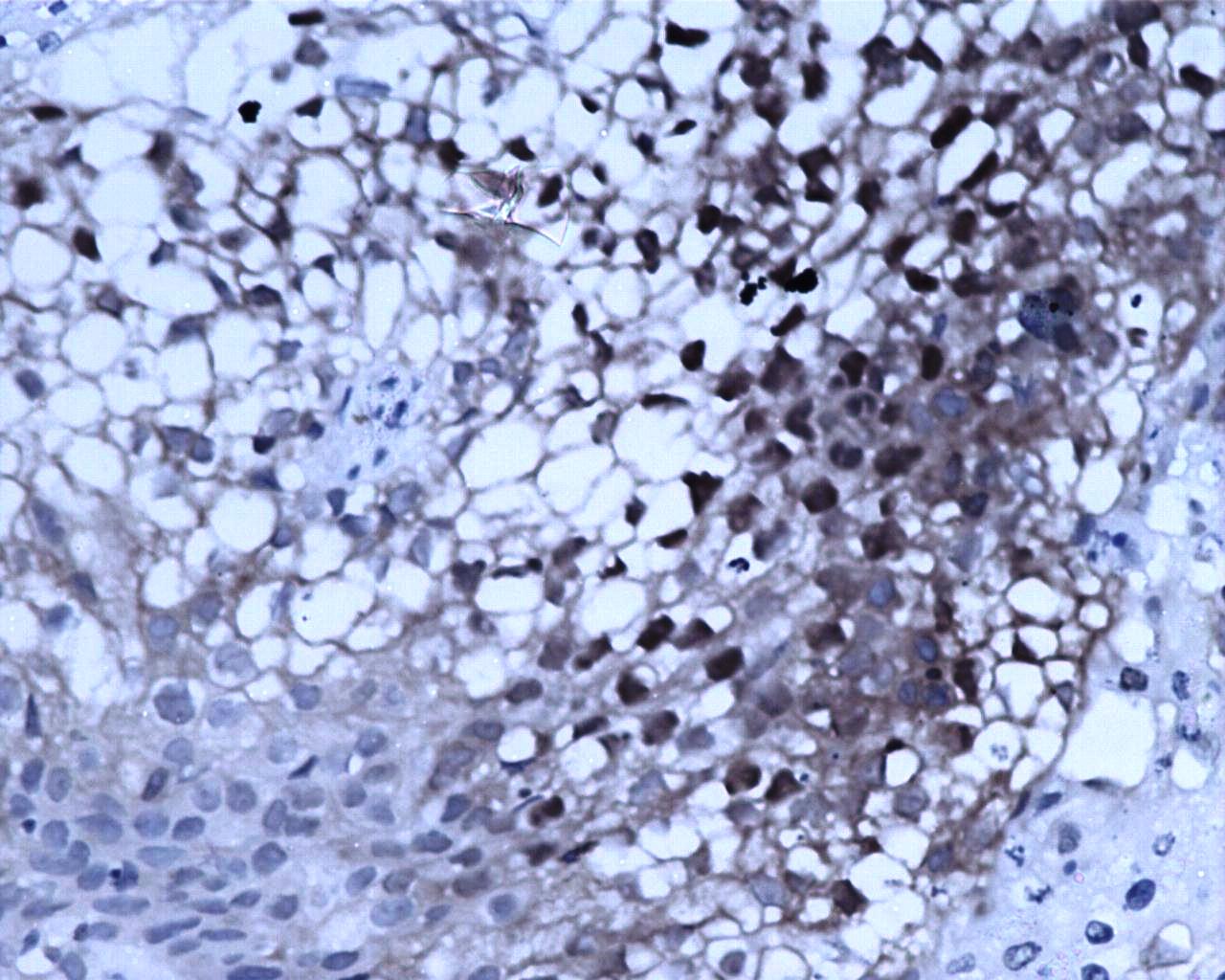

Supplement: Supplemental Information 7 — (E) Immunostaining with RelA (p65) showed nuclear positivity (Brown colour) as indicated by arrows IHC × 200. [file peerj-06-5563-s007.jpg]

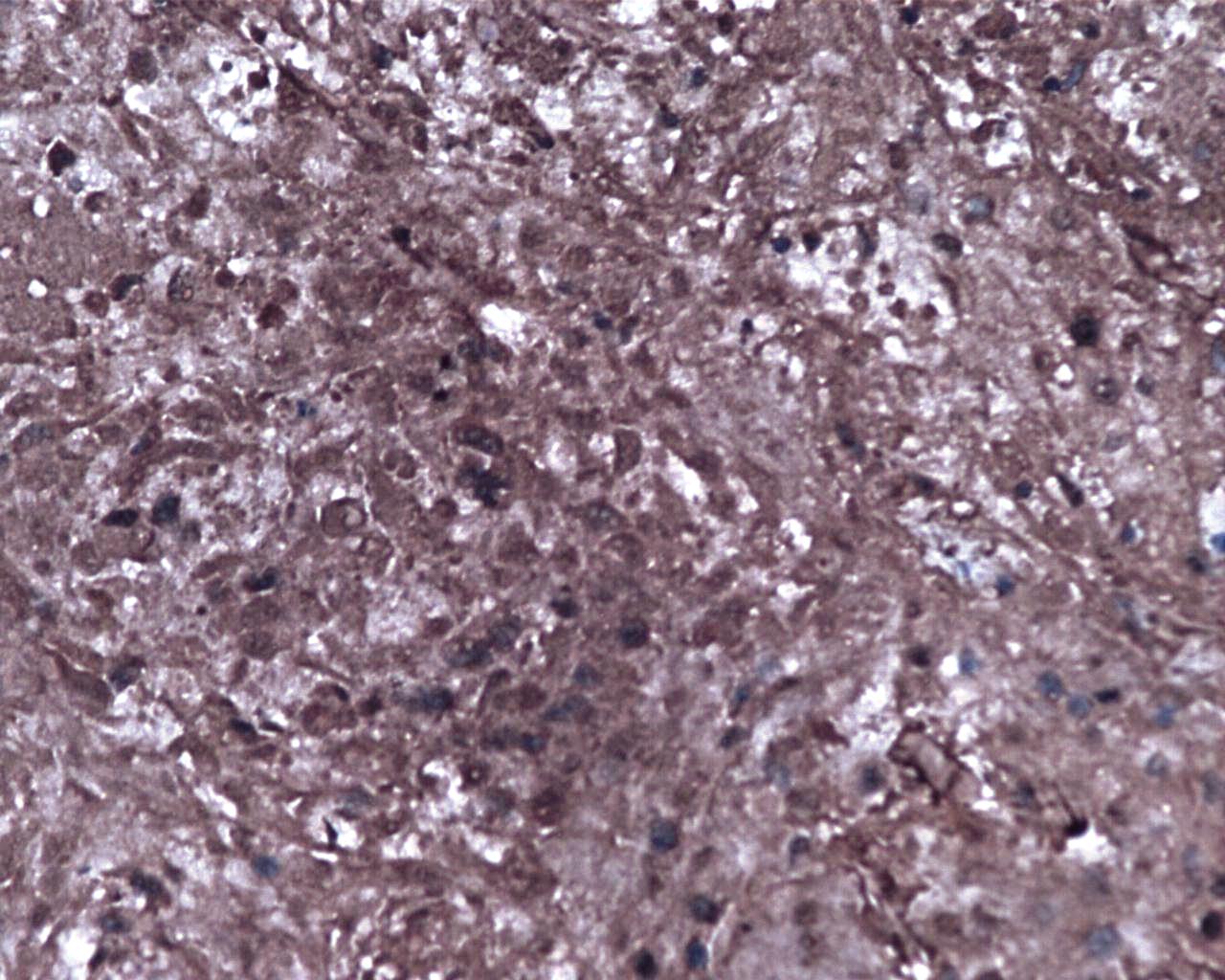

Supplement: Supplemental Information 8 — (F) NF-κBp50 showed few nuclear and cytoplasmic positivity in squamous regions which are indicated by arrows IHC × 200. [file peerj-06-5563-s008.jpg]

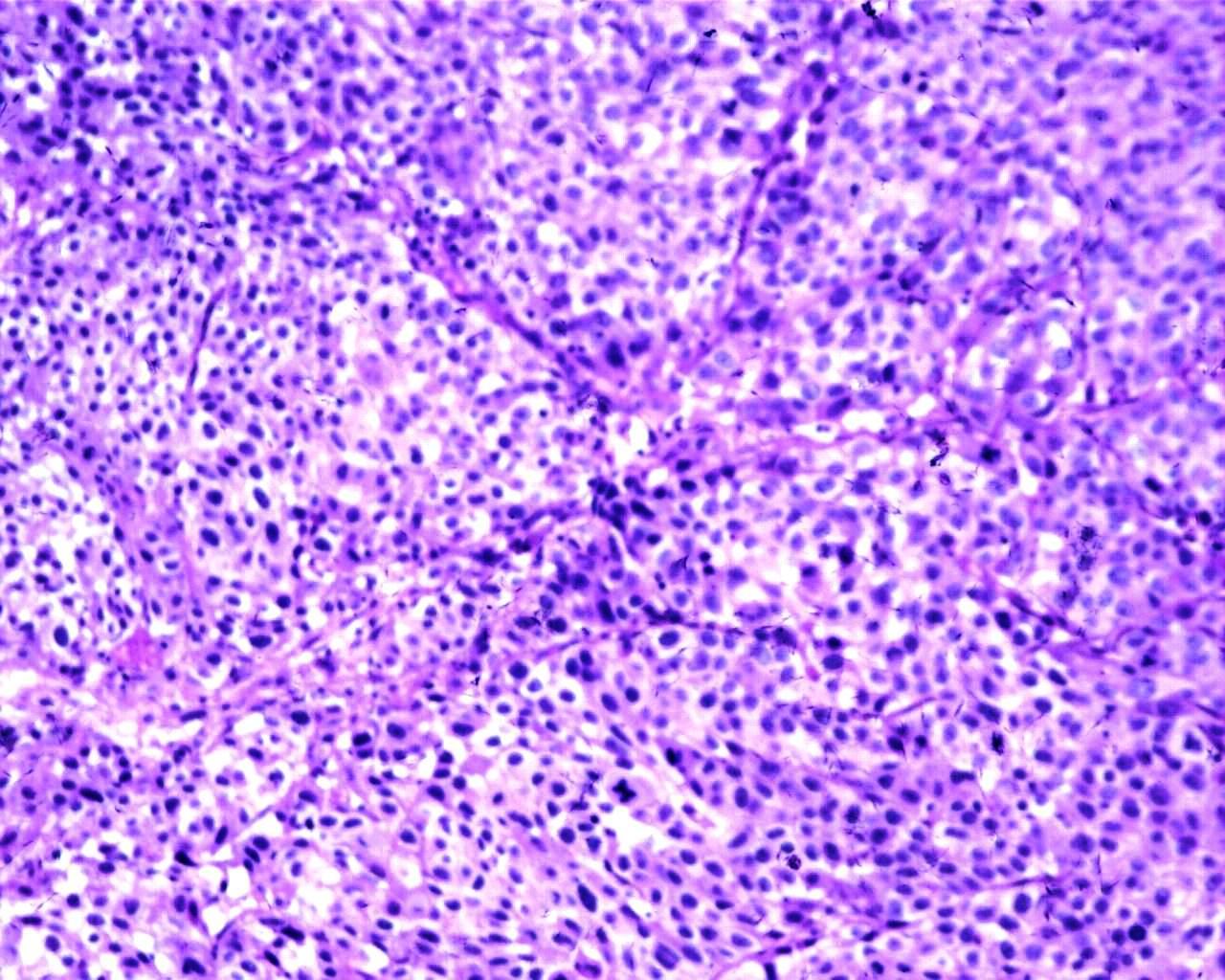

Supplement: Supplemental Information 9 — (G) High grade invasive urothelial carcinoma H&E × 100. [file peerj-06-5563-s009.jpg]

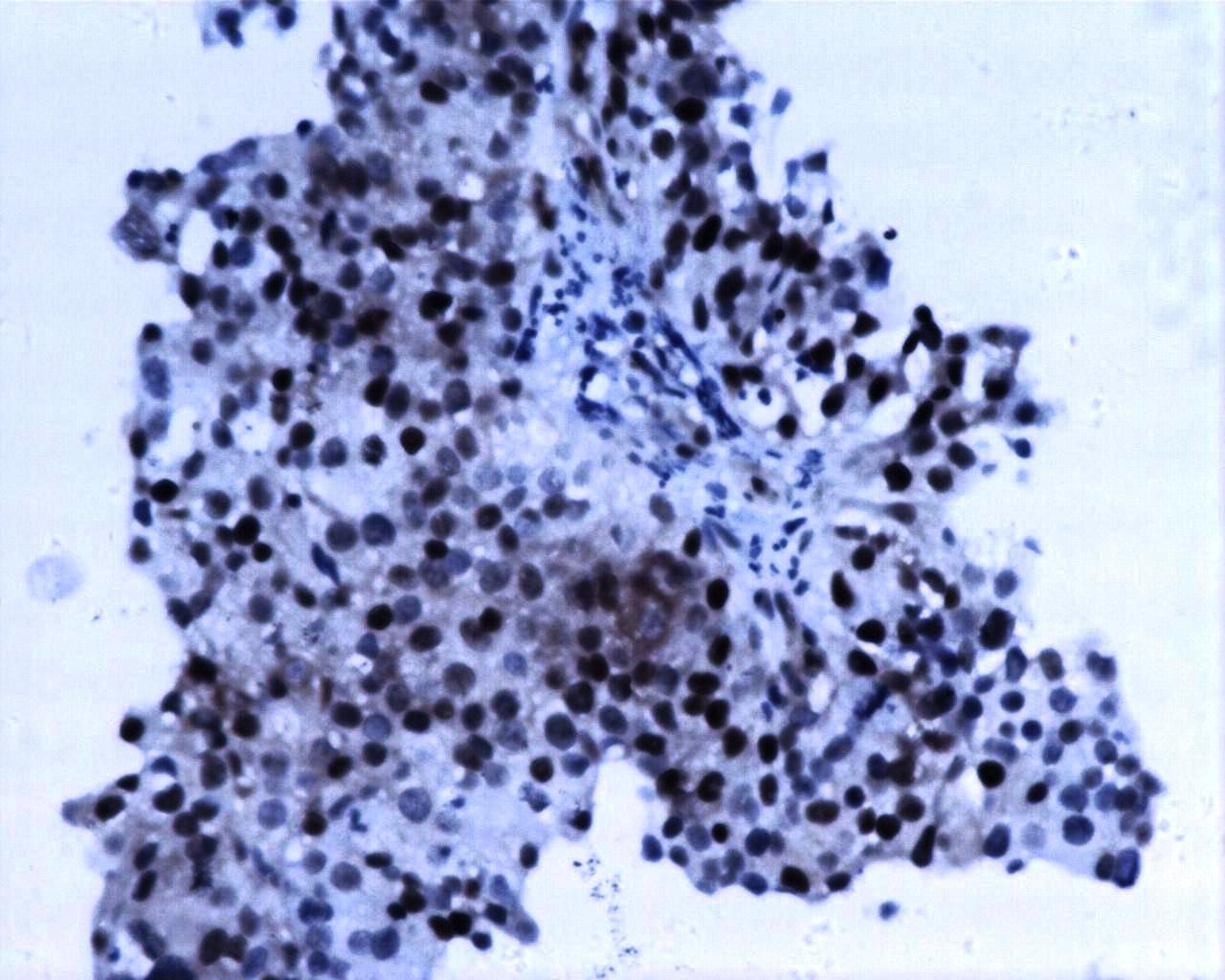

Supplement: Supplemental Information 10 — (H) Immunostaining with RelA (p65) showed nuclear positivity (Brown colour) as indicated by arrows IHC × 200. [file peerj-06-5563-s010.jpg]

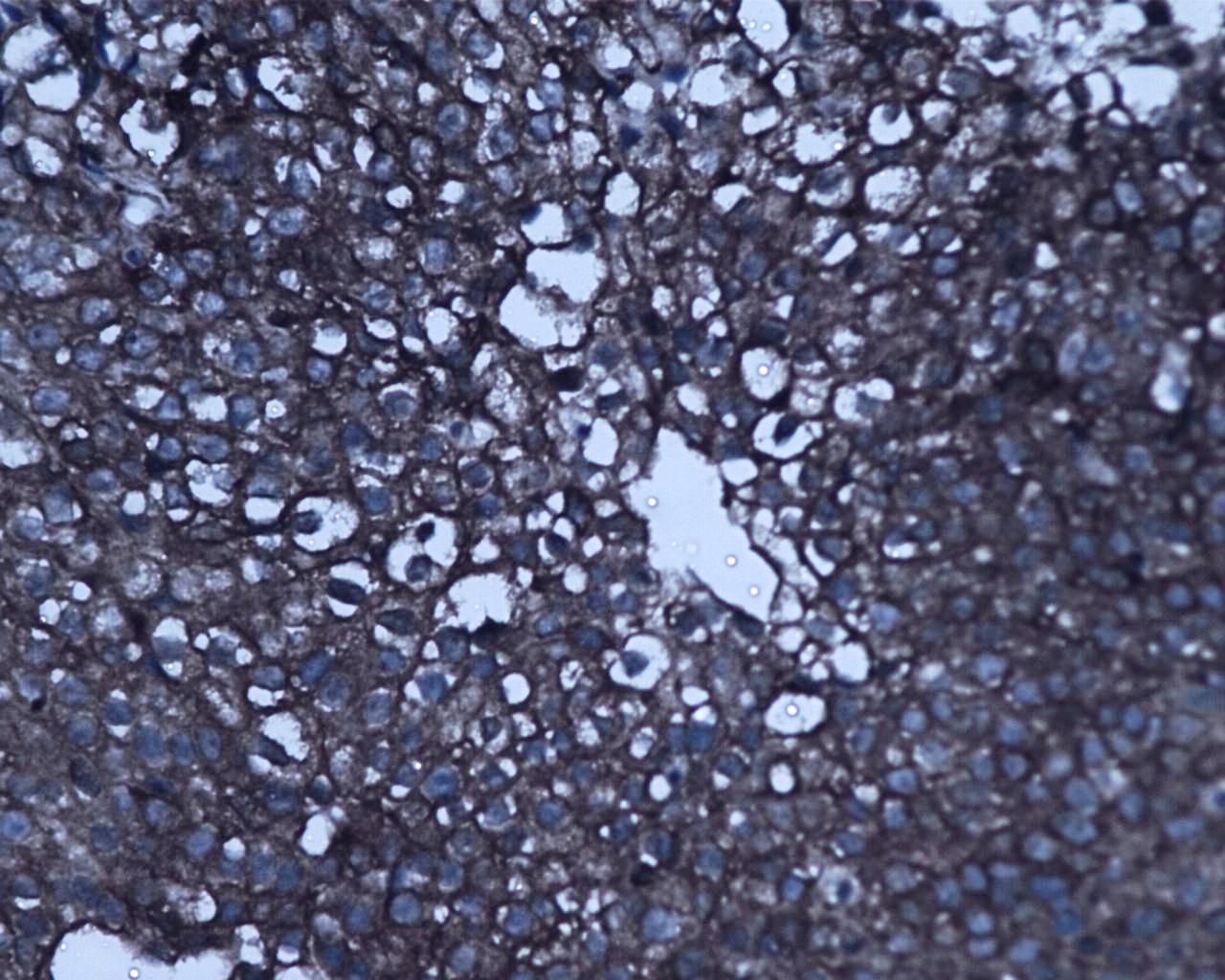

Supplement: Supplemental Information 11 — (I) NF-κBp50 showed extensive cytoplasmic positivity as indicated by arrows IHC × 200. [file peerj-06-5563-s011.jpg]

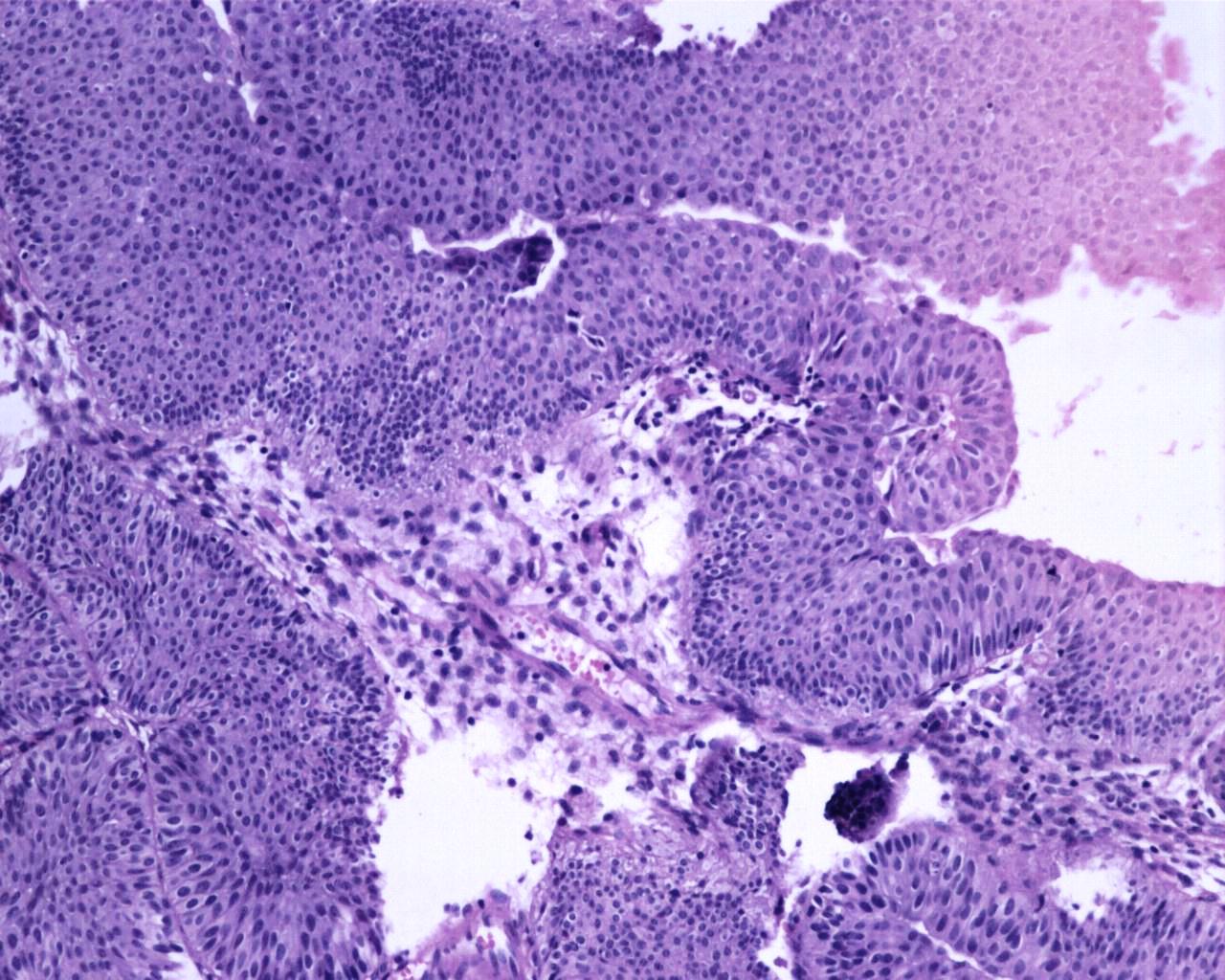

Supplement: Supplemental Information 12 — (A) Low grade papillary urothelial carcinoma H&E × 100. [file peerj-06-5563-s012.jpg]

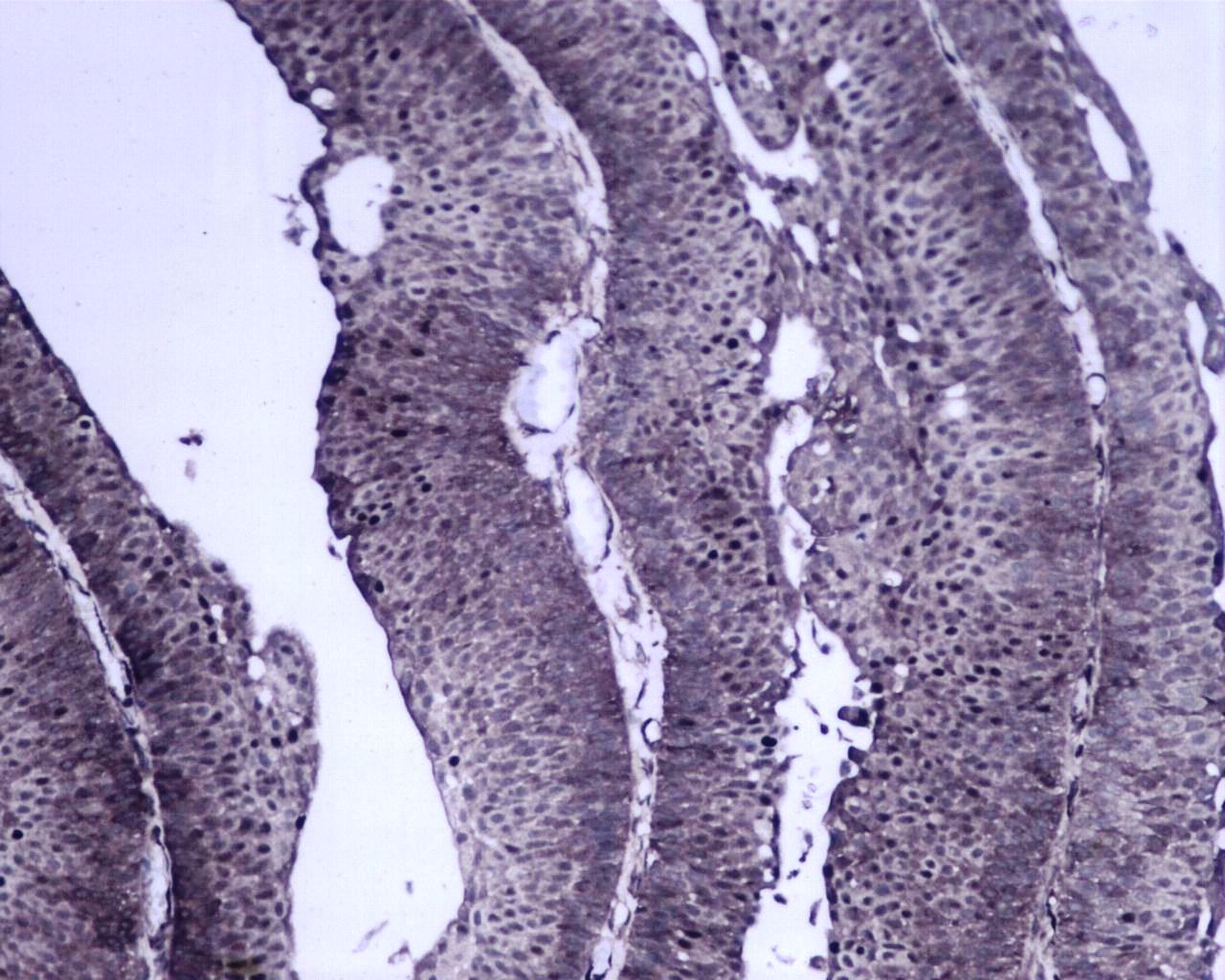

Supplement: Supplemental Information 13 — (B) Immunostaining with RelA (p65) showed nuclear positivity (Brown colour) IHC × 100. [file peerj-06-5563-s013.jpg]

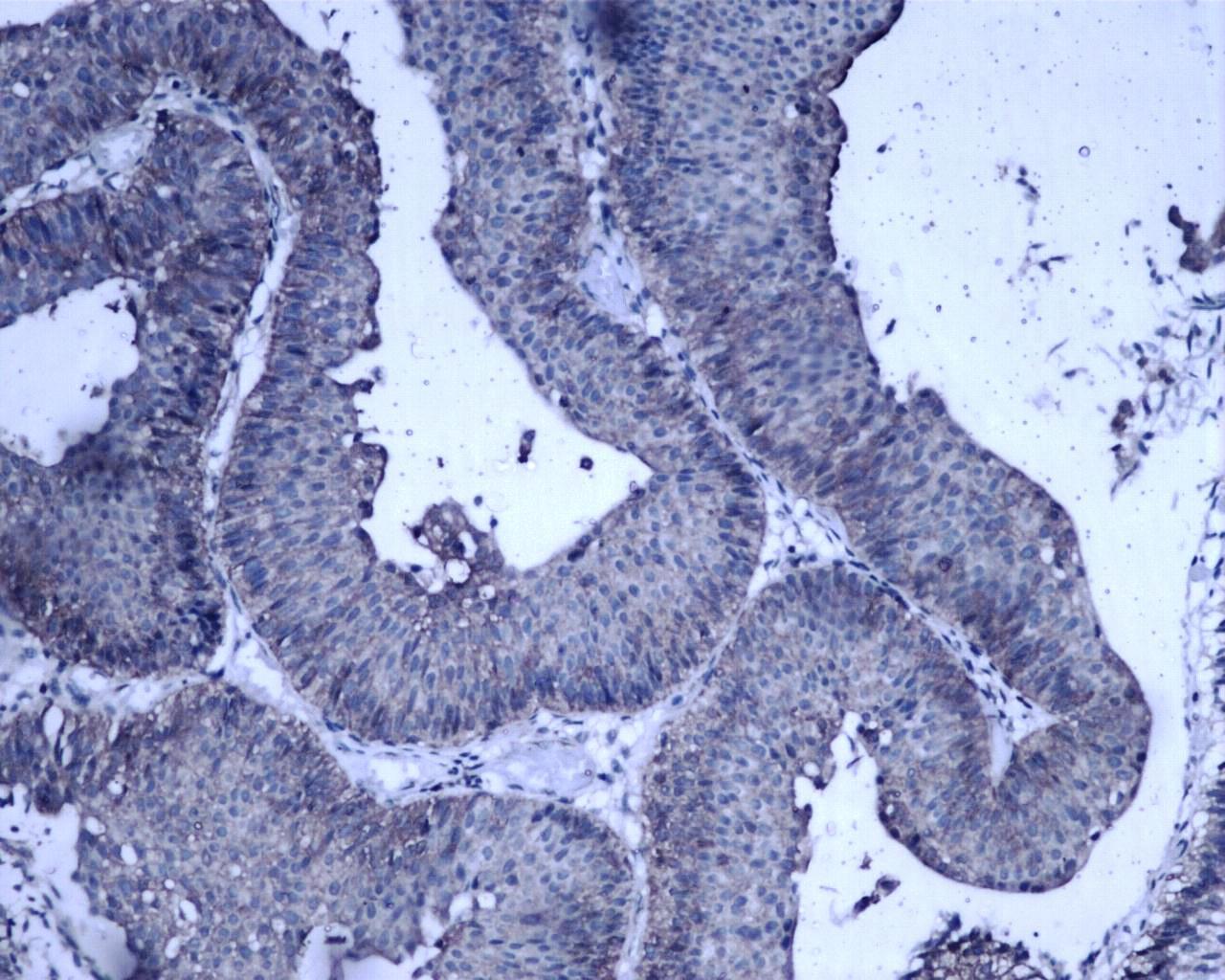

Supplement: Supplemental Information 14 — (C) NF-κBp50 showed extensive cytoplasmic positivity IHC × 100. [file peerj-06-5563-s014.jpg]

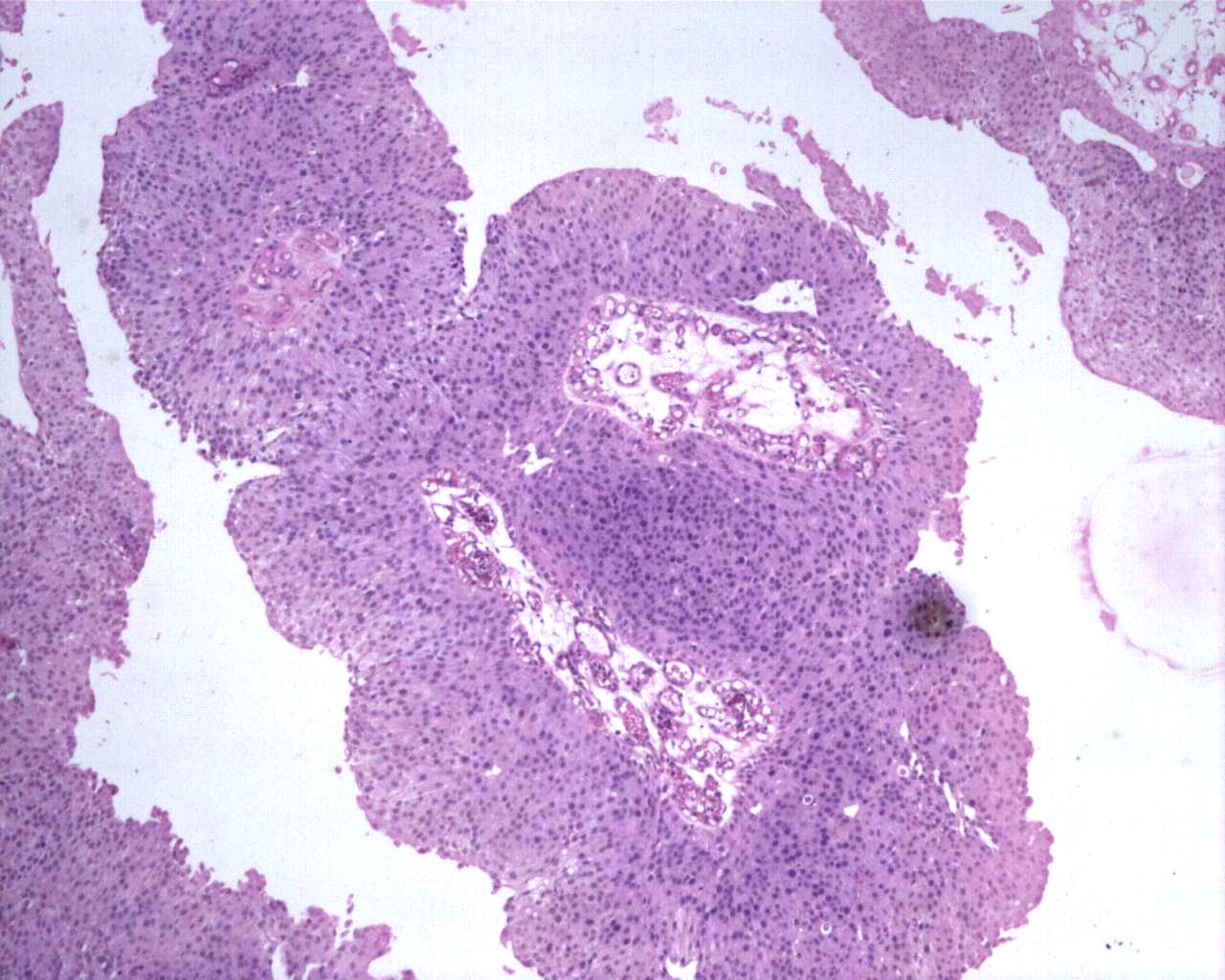

Supplement: Supplemental Information 15 — (D) Low grade papillary urothelial carcinoma with tumor infiltration H&E × 40. [file peerj-06-5563-s015.jpg]

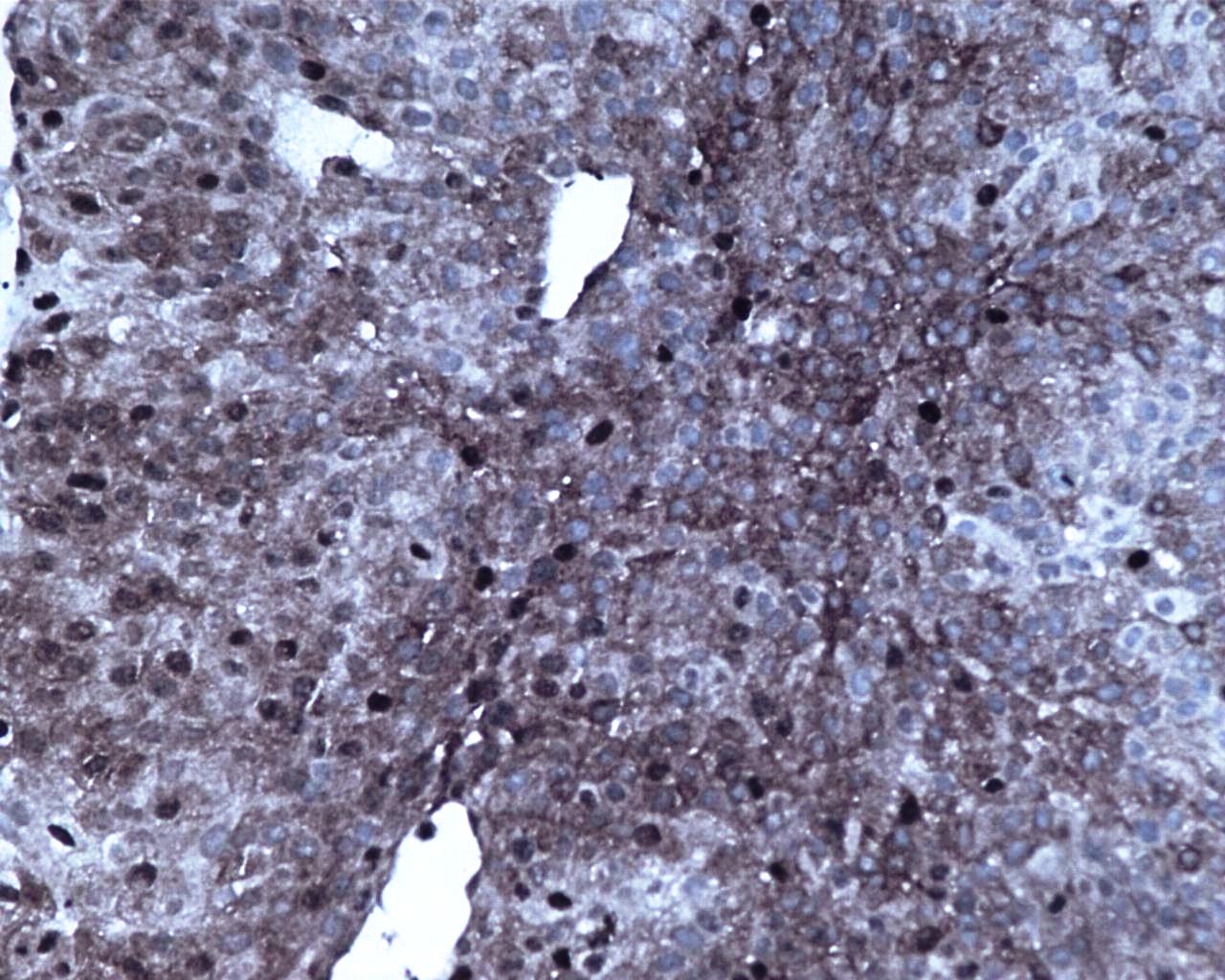

Supplement: Supplemental Information 16 — (E) Immunostaining with RelA (p65) showed nuclear positivity (Brown colour) IHC × 200. [file peerj-06-5563-s016.jpg]

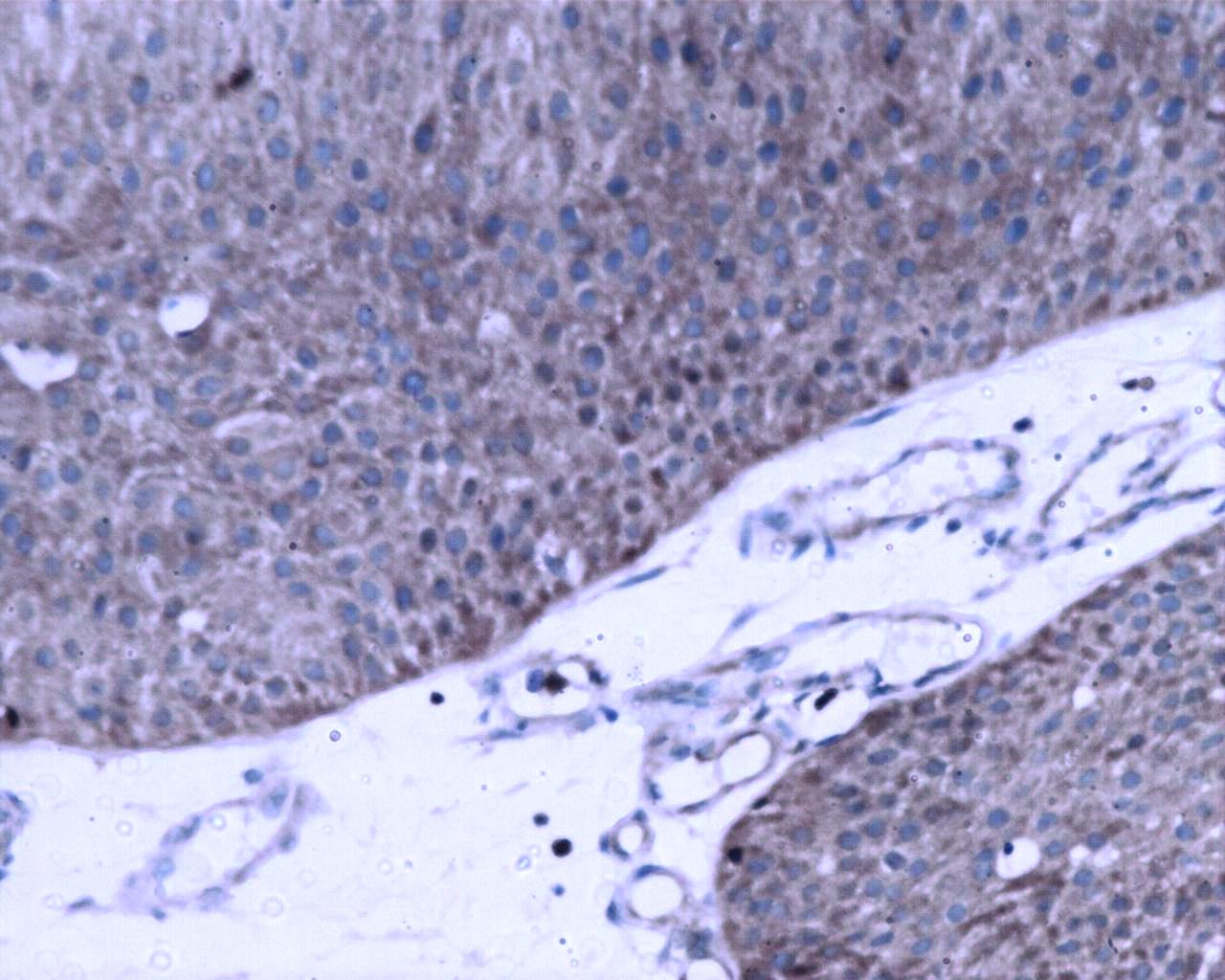

Supplement: Supplemental Information 17 — (F) NF-κBp50 showed moderate cytoplasmic positivity IHC × 200. [file peerj-06-5563-s017.jpg]

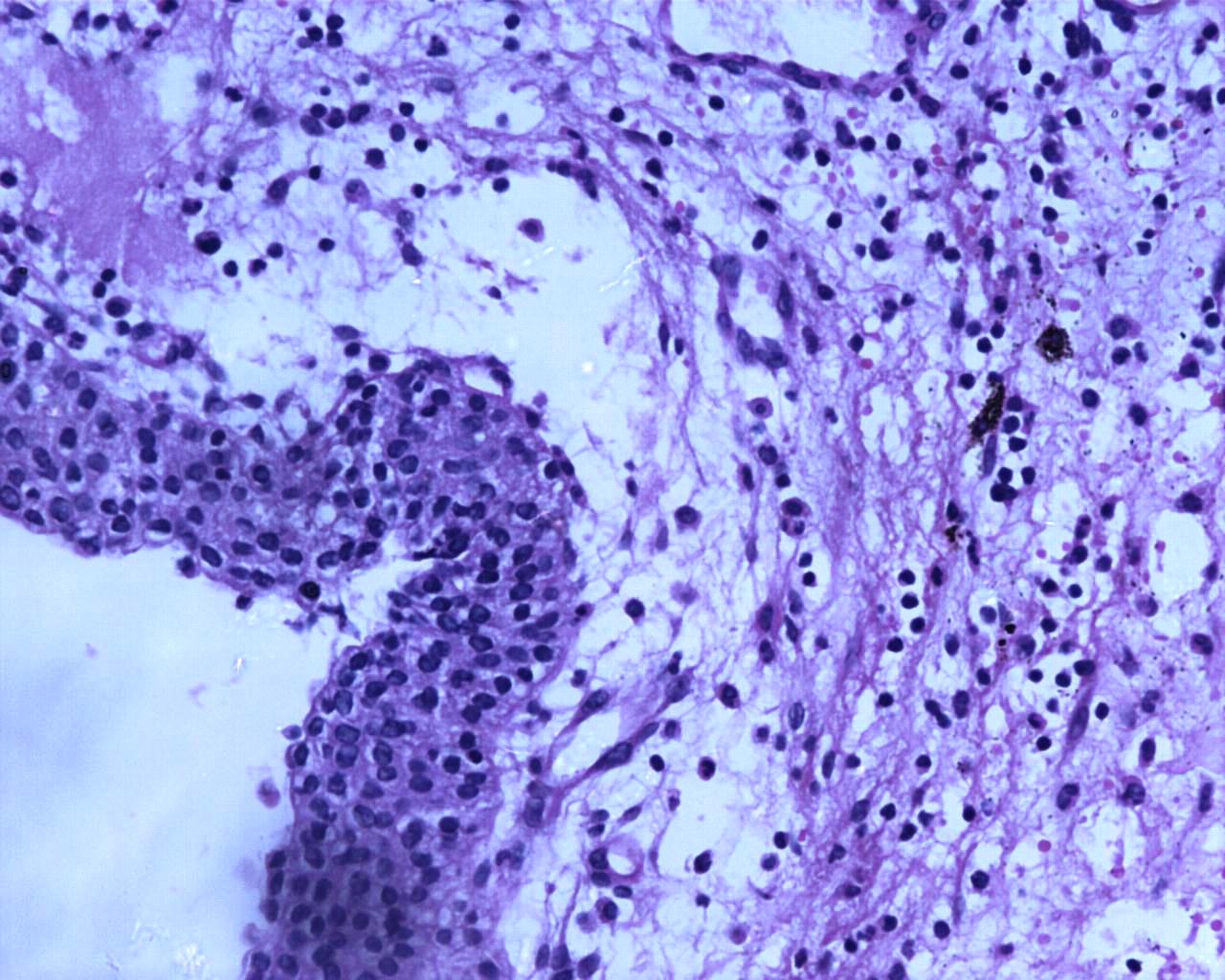

Supplement: Supplemental Information 18 — (G) Normal urothelium H&E × 200. [file peerj-06-5563-s018.jpg]

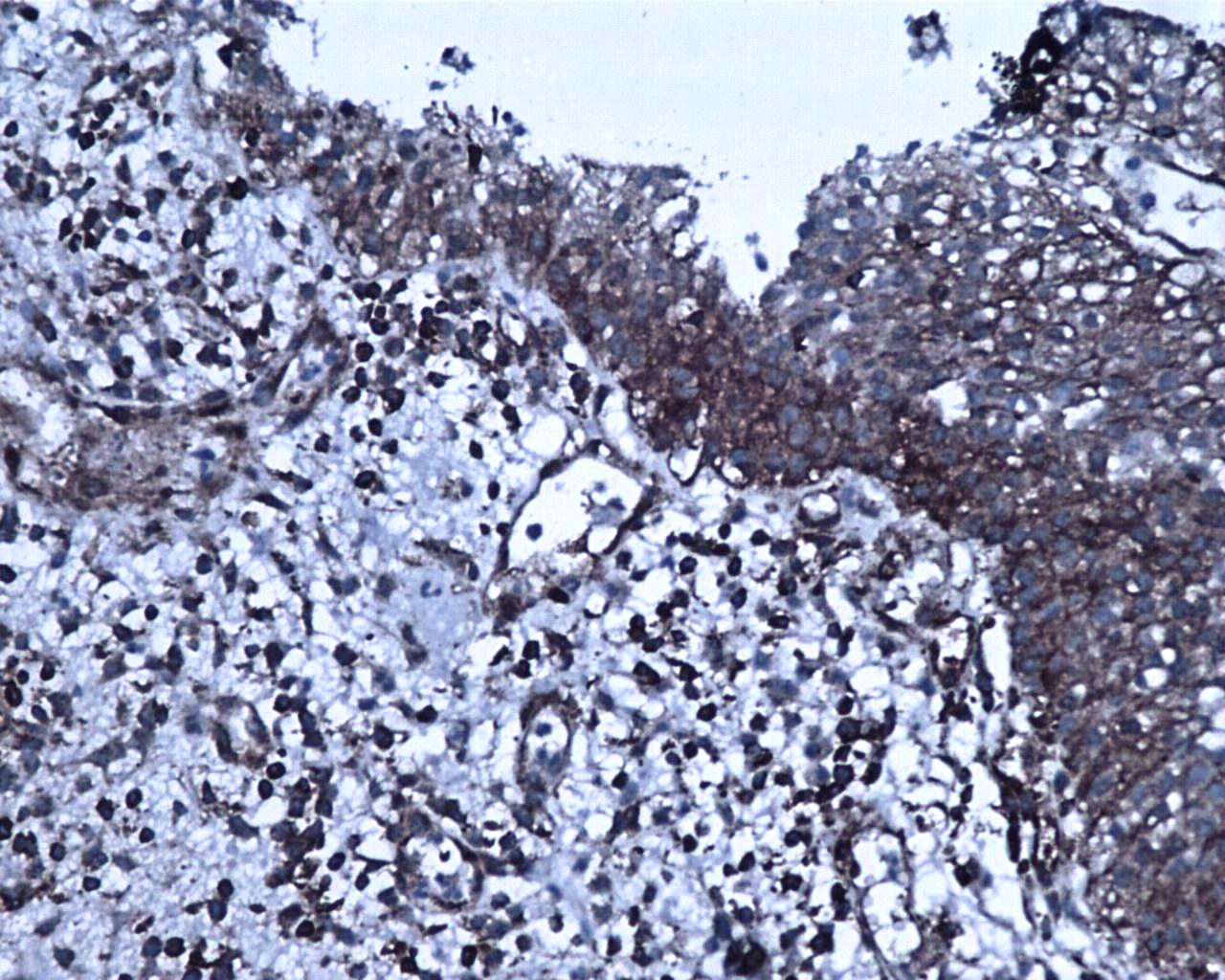

Supplement: Supplemental Information 19 — (H) Immunostaining with RelA (p65) showed faint to moderate cytoplasmic positivity (Brown colour) IHC × 200. Note: Lymphocytes (in-built control) have taken up stain indicated by arrows. [file peerj-06-5563-s019.jpg]

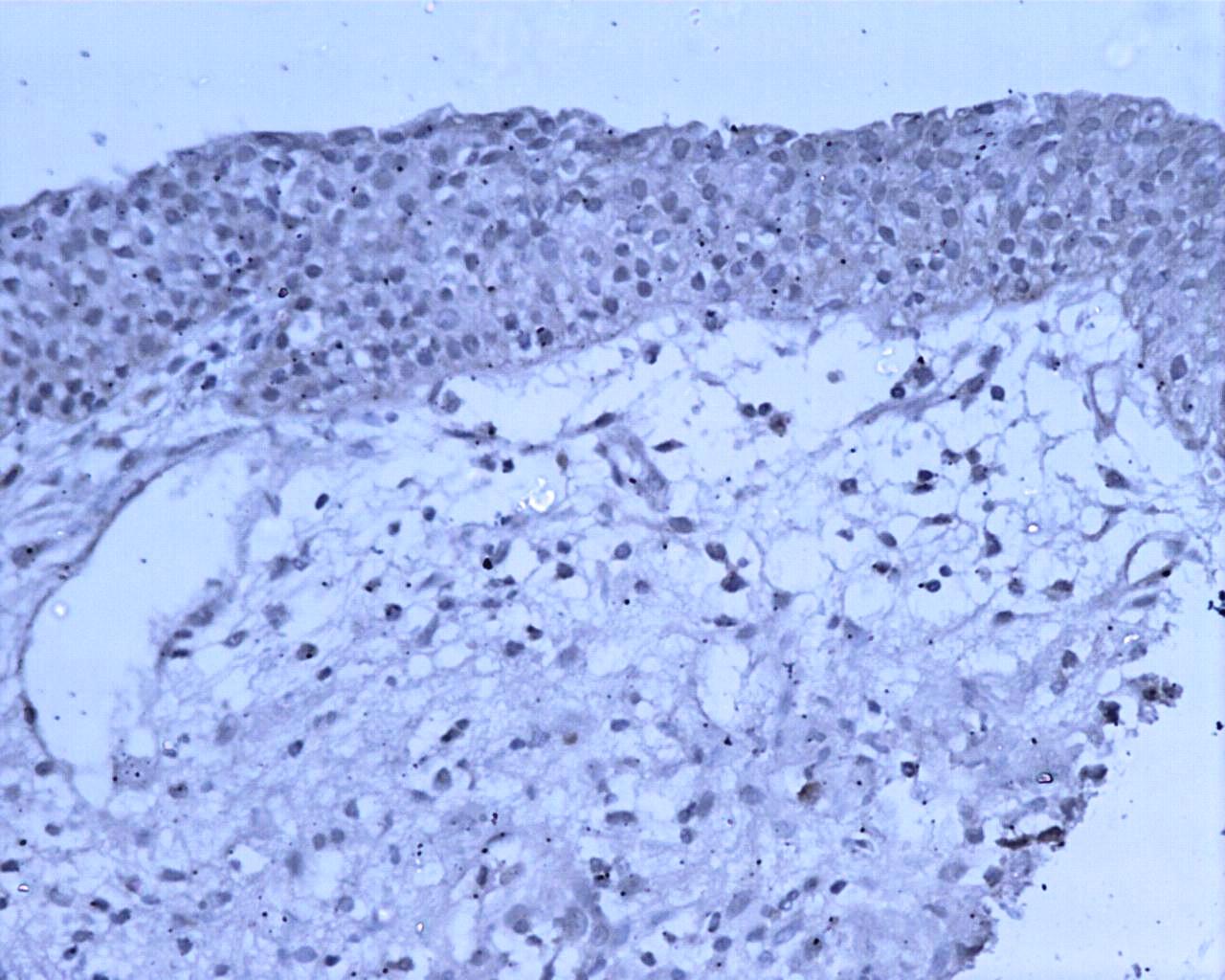

Supplement: Supplemental Information 20 — (I) NF-κB p50 showed moderate cytoplasmic positivity indicated by arrow IHC × 200. [file peerj-06-5563-s020.jpg]

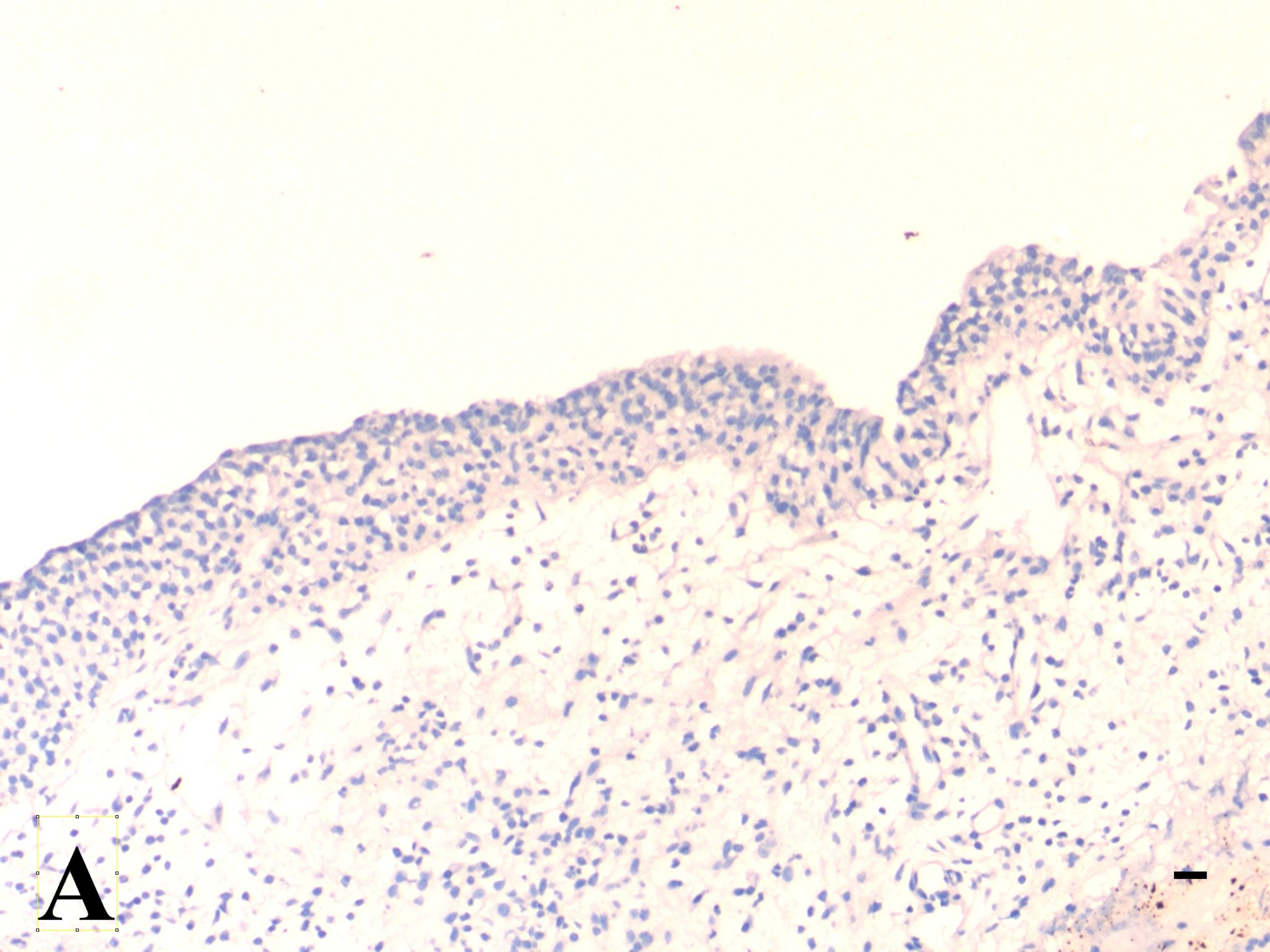

Supplement: Supplemental Information 21 — A, RelA subunit staining is a Negative control (Reagent)—No primary antibody was applied on sections [file peerj-06-5563-s021.jpg]

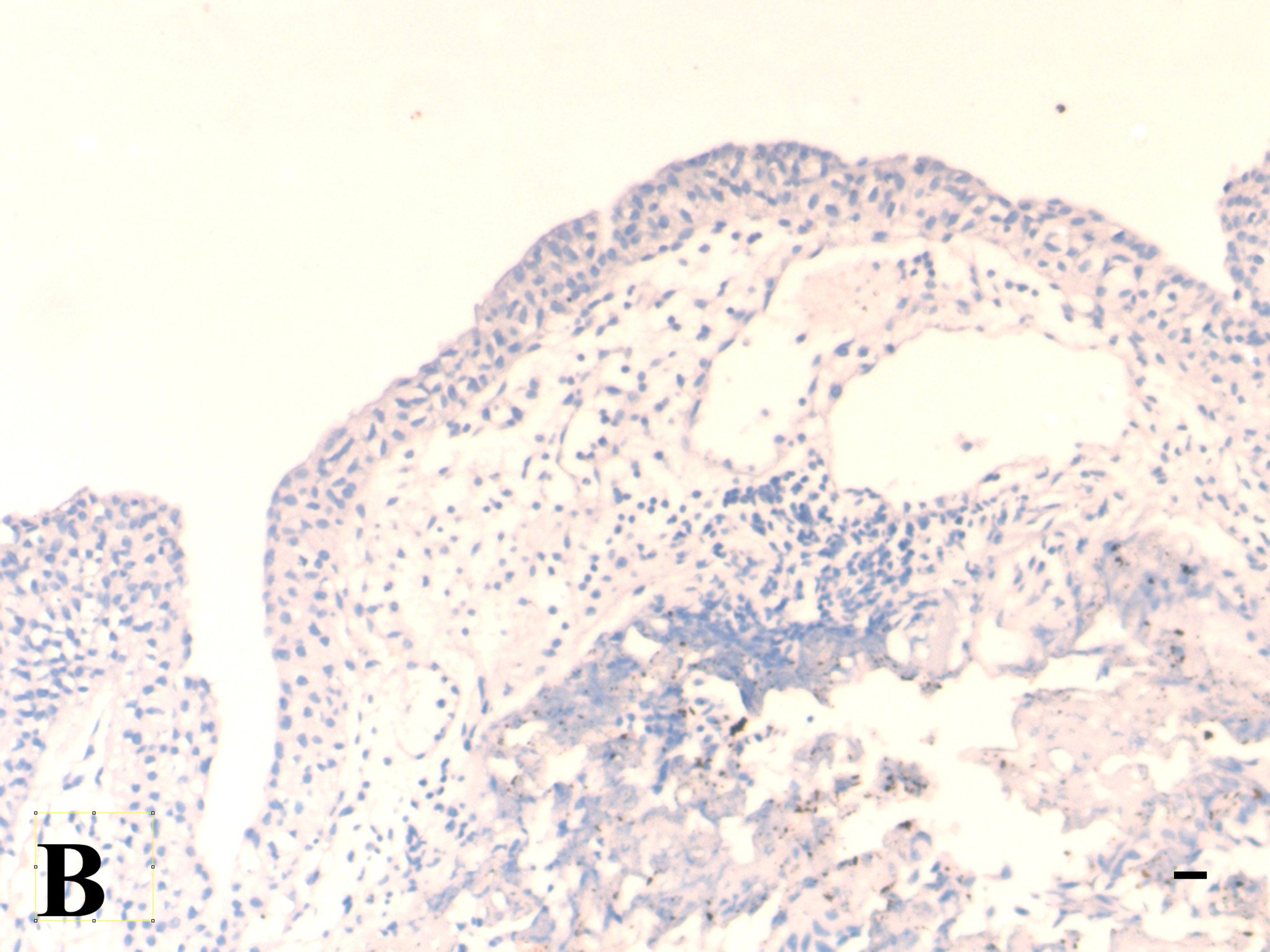

Supplement: Supplemental Information 22 — B, p50 subunit staining is a Negative control (Reagent)—No primary antibody was applied on sections [file peerj-06-5563-s022.jpg]
